# Impact of scapula anatomical landmark positioning on scapular orientation using CT-based 3-dimensional models: an intraobserver repeatability and interobserver reproducibility study

**DOI:** 10.1016/j.jseint.2024.09.027

**Published:** 2024-10-18

**Authors:** Florent Moissenet, Sana Boudabbous, Nicolas Holzer

**Affiliations:** a Biomechanics Laboratory, Faculty of Medicine, Geneva University Hospitals and University of Geneva, Geneva, Switzerland; b Kinesiology Laboratory, Geneva University Hospitals and University of Geneva, Geneva, Switzerland; c Department of Radiology, Geneva University Hospitals, Geneva, Switzerland; d Department of Surgery, Geneva University Hospitals, Geneva, Switzerland

**Keywords:** Anatomical landmarks, Surface model, Scapulothoracic joint, Surgical preoperative planning, Reverse total shoulder arthroplasty, Shoulder

## Abstract

**Hypothesis:**

The primary objective of this study was to assess intraobserver repeatability and interobserver reproducibility of the 3-dimensional (3D) coordinates of a set of scapula anatomical landmarks obtained by manual positioning on conventional supine computed tomography (CT) scan–based scapula surface models. The secondary objective was to assess intraobserver repeatability and interobserver reproducibility of the resulting 3D scapular orientation. It was hypothesized that the 3D scapular orientation reliability would be better or similar than established reliability of intraoperative baseplate positioning (ie, version and inclination) in reverse total shoulder arthroplasty.

**Methods:**

Three anatomical landmarks, ie, acromial angle, inferior angle, and trigonum spinae, were manually positioned on 81 scapula surface models using updated landmark definitions. These models were obtained by the segmentation of CT scan images acquired from patients undergoing elective reverse total shoulder arthroplasty procedures at the Department of Surgery at Geneva University Hospitals between May 2022 and December 2023. This procedure was repeated 3 times per scapula by 3 independent observers. A set of parameters corresponding to the 3D landmark coordinates (expressed in an average scapula coordinate system) and the 3D scapular orientation (expressed as 3 angles related to the retraction/protraction, lateral/medial rotation, and internal/external rotation) were computed. Intraobserver repeatability and interobserver reproducibility were assessed for each of these parameters using an intraclass correlation coefficient (ICC) as a relative reliability metric. Standard error of measurement (SEM) was also reported as an absolute reliability metric.

**Results:**

Intraobserver ICC and interobserver ICC ranged from poor to moderate for acromial angle, poor to excellent for inferior angle, and poor to excellent for trigonum spinae. However, low SEM, ranging from 0.4 mm to 2.4 mm for intraobserver repeatability and from 0.2 mm to 1.8 mm for interobserver reproducibility, was obtained for every coordinate of each anatomical landmark. This results in poor to moderate intraobserver ICC and interobserver ICC for scapula angular orientation. Again, low SEM, ranging from 0.4° to 0.8° for intraobserver repeatability and 0.4° to 0.6° for interobserver reproducibility, was obtained, arguing for reliable measurements.

**Discussion:**

This study demonstrates that manual positioning of the 3 scapula anatomical landmarks recommended by the International Society of Biomechanics can be performed in a reliable manner across measures and observers on surface bone models obtained from CT scan images. However, a clear and standardized definition of these landmarks is needed to ensure consistency across measurements.

Determination of scapular 3-dimensional (3D) orientation is a frequent requisite for shoulder girdle investigations and procedures. The importance of considering the scapulothoracic joint in the reverse total shoulder arthroplasty (rTSA) planning process has recently been highlighted by Moroder et al.^21^, ^22^, ^23^ In particular, the scapular orientation related to the thorax was shown as having an influence in the determination of the optimal humeral component retrotorsion avoiding notching and maximizing range of motion (ROM).

To date, rTSA planning software focus solely on the glenohumeral joint by simulating humeral motions around the axes of the scapula coordinate system (CS).^5^ However, in a recent study, Thomas et al reported no significant correlations between postoperative thoracohumeral ROMs measured clinically and preoperative glenohumeral ROMs predicted using a planning software.^30^ These authors suggested that this gap could be filled, above other aspects, by considering the resting scapular posture. Introduction of the resting scapular posture (ie, the orientation of the scapula in relation to the thorax) in the rTSA planning process might thus be crucial step toward realistic projections.^21^, ^22^, ^23^

Currently, preoperative planning is commonly based on computed tomography (CT) scan images obtained in supine position. The resulting scapular orientation related to the thorax is then different than in standing position.^17^ Defining the scapular orientation in standing posture appears thus to be a first key step toward improving the rTSA planning process. This measurement has been addressed through several approaches in the literature. On one hand, motion capture systems have been used together with cutaneous markers,^26^ calibrated anatomical systems technique like method with calibrated stylus,^1^ or anatomical palpator.^25^ On the other hand, medical imaging systems have been used through different modalities such as vertical CT scan,^29^ vertically open magnetic resonance imaging (MRI),^24^ biplanar X-rays,^3^ or ultrasounds.^2^ Except vertical CT scan and vertically open MRI approaches allowing bone segmentation based on acquired images, all other previously mentioned approaches require a 3D bone registration process. Through this process, a bone surface model, obtained from previously acquired CT scan or MRI images, is positioned in space. This can be done using a paired-point technique (ie, rigid transformation) when only a set of anatomical landmarks can be recorded in standing posture (eg, using motion capture systems), and/or a paired-surface technique (ie, iterative closest point algorithm) when at least 1 partial bony surface can be recorded (eg, using ultrasounds).^11^ Before introducing such approaches in the preoperative rTSA planning, it is essential to assess their reliability and accuracy in scapular orientation measurement. This study will focus exclusively on the first approach, which requires the use of anatomical landmarks. Above other issues, landmarks positioning on bone surface models is a crucial step. Manual positioning represents the conventional approach while being time-consuming compared to semiautomatic or automatic approaches.^6^^,^^18^ Still, manual positioning remains the ground truth used to train a deep-learning network such as a convolutional neural network framework.^6^ Without reliable and accurate manual positioning, resulting semiautomatic or automatic tools might not generate relevant data. To the best of our knowledge, very few studies have assessed the reliability of anatomical landmark manual positioning. Intraobserver repeatability and interobserver reproducibility was examined by Jacquot et al on 3D positioning of the trigonum spinae (TS), the inferior angle (IA), and the glenoid center on 82 scapulae.^12^ Similarly, Gilliland et al examined 3D positioning reliability of the superior, inferior, and lateral base of coracoid, supraglenoid tubercle, TS, top of scapular notch, centroid of coracoid base, acromion lateral, and coracoid tip on 10 scapulae.^10^ Both studies reported moderate to excellent intraclass correlation coefficient (ICC). However, 2 primary limitations can be highlighted in these studies. First, landmark selection was not suitable for all approaches using skin sensors to estimate scapular orientation. In this respect, the 3 landmarks proposed by the International Society of Biomechanics (ISB), ie, TS, IA, and acromial angle (AA), might be more relevant. To date, reliability of the AA landmark manual positioning remains unreported. Second, the primary interest is not to assess 3D landmark coordinates but to estimate their impact on 3D scapular orientation. To date, this impact remains unqualified. To provide significant support to the surgical procedure, the resulting scapular orientation reliability should be better or similar than established reliability of intraoperative baseplate positioning (ie, version and inclination) in rTSA, ie, 5.9 ± 3.5° and more using a conventional approach, and 1.9 ± 1.9° and more using navigation.^13^^,^^31^

The primary objective of this study was to assess the intraobserver repeatability and interobserver reproducibility of the 3D coordinates of ISB scapula landmarks (ie, TS, IA, and AA) obtained by manual positioning on conventional supine CT scan based scapula surface models. The secondary objective was to assess intraobserver repeatability and interobserver reproducibility of the resulting 3D scapular orientation. It was hypothesized, using a clear landmark definition, that the data dispersion observed on scapular orientation would be lower than the intraoperative error observed on baseplate positioning in rTSA. It was hypothesized that the 3D scapular orientation reliability would be better or similar than established reliability of intraoperative baseplate positioning (ie, version and inclination) in rTSA.

## Materials and methods

### Study design and population

This monocentric retrospective cohort study was approved by the Cantonal Research Ethics Commission of Geneva (CER 2019-00069). CT scan images from patients undergoing elective rTSA procedures at the Department of Surgery at Geneva University Hospitals between May 2022 and December 2023 were retrieved. All patients planned for surgery during this period were included. Data related to partial scapula view were excluded. All participants provided written informed consent.

### Data collection

**Figure 1:**
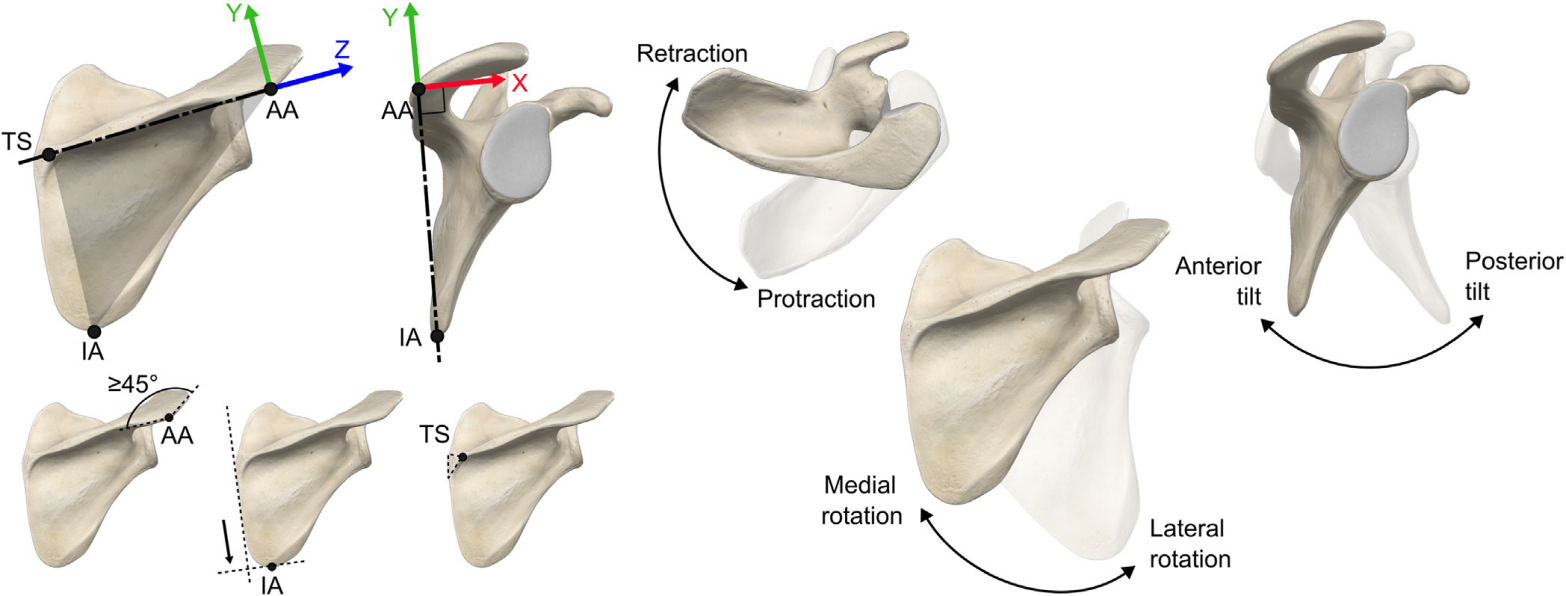
Anatomical landmarks and coordinate system of the scapula used in this study. *AA*, acromial angle; *IA*, inferior angle; *TS*, trigonum spinae.

All CT scan images were acquired using our standard protocol for rTSA. Briefly, this protocol consists in short spiral axial scans documenting the affected shoulder using a 24-row CT unit (Somatom Drive computed tomography; Siemens Healthcare, Forchheim, Germany). The following scan parameters were used: field of view, 30 cm; matrix 512 × 512; slice thickness, 1.0 mm; and interval of reconstruction, 1.0 mm, 120 kVp, and 120 mA. Scapula surface models were segmented using a semiautomatic method (Mimics, Materialise, Leuven, Belgium) and provided by the implant manufacturer (Medacta Intl., Castel San Pietro, Switzerland through our normal preoperative 3D planning for rTSA. These models were stored in stereolithography files, a widely used file format native to prepare surface models. The 3 scapula landmarks proposed by the ISB^32^ (ie, TS, IA, and AA, Fig. 1) were then manually positioned using 3D Slicer (5.6.1; 3D Slicer, Earth, TX, USA)^9^ following the procedure reported in Figure 1 and Table I.

**Table I:** Anatomical landmarks definition and manual positioning strategy.

ID	Definition	Positioning strategy
AA	Acromial angle	First angle higher than 45° from the lateral edge of the acromion
IA	Inferior angle	Most distal point of the scapula along the medial edge of the scapula
TS	Root of the scapular spine	Apex of the root triangle, closest to the scapular spine

*AA*, acromial angle; *IA*, inferior angle; *TS*, trigonum spinae.

This procedure was repeated 3 times per scapula by 3 independent observers (FM, SB, NH). A randomized list of 243 (81 scapulae, 3 measures) models with a new identifier was generated to avoid any learning. Prior training was carried out on another dataset^19^ of 10 scapulae to clarify landmarks definition and manual positioning strategy (Fig. 1, Table I).

All observers were experienced and involved in the 3D imaging–assisted surgery process. 3D landmark coordinates were expressed in the imaging CS at this stage and stored, for each scapula, observer, and measure, in a comma-separated values file, a simple text file that stores tabular data in a plain text format and separate them by a comma.

### Data processing

A custom Matlab program (R2022b; Mathworks, Natick, MA, USA) was used to compute the 3D landmark coordinates and the 3D scapular orientation.

The 3D landmark coordinates were expressed in an average scapula CS to allow comparison across observers and measures. For each scapula, the mean landmarks mIA, mAA, and mTS related to IA, AA, and TS were computed across observers and measures. The average scapula CS was then defined by setting its axes $X_{acs}$, $Y_{acs}$, and $Z_{acs}$ following ISB recommendations^32^: $X_{acs}$ was the unitary vector corresponding to the normal to the plane defined by mAI, mAA, and mTS, pointing anteriorly, $Z_{acs}$ was the unitary vector along the line crossing mTS and mAA, pointing medially, and $Y_{acs}$ was the unitary vector resulting from the cross product of $Z_{acs}$ and $X_{acs}$, pointing superiorly. The resulting rotation matrix $R_{acs}^{ics}=\left[X_{acs}\;Y_{acs}\;Z_{acs}\right]$ was used to express each 3D landmark coordinates in the average scapula CS: $P_{acs}^{i}{=\text{inv}(R}_{acs}^{ics})\cdot P_{ics}^{i}$, where $P_{ics}^{i}$ are the coordinates of the landmark *i* in the imaging CS (implicitly defined in the coordinates of each landmark in the surface model) and $P_{acs}^{i}$ are the coordinates of the landmark *i* in the average scapula CS. Hence, for each scapula, the 3D landmark coordinates obtained for every observer and measure were expressed in the same CS.

The 3D scapular orientation was expressed as 3 angles related to the retraction/protraction, lateral/medial rotation, and internal/external rotation (Fig. 1). As ISB thorax landmarks were not available in CT scan images, the average scapulothoracic joint orientation reported by Ludewig et al^16^ during a relaxed standing posture was used instead defining a thorax CS: 41.1° of protraction, 5.4° of medial rotation, and 13.5° of anterior tilt. The resulting rotation matrix $R_{acs}^{tcs}$, combining these 3 successive rotations, was applied to the 3D landmark coordinates: $P_{tcs}^{i}=R_{acs}^{tcs}\cdot P_{acs}^{i}$. A specific scapula CS was then defined, for each scapula, observer and measure, following the same rules than for the average scapula CS to set $X_{scs},Y_{scs}$, and $Z_{scs}\text{.}$ The resulting rotation matrix $R_{scs}^{tcs}=\left[X_{scs}\;Y_{scs}\;Z_{scs}\right]$ was finally used to compute the 3D scapular orientation around the axes of the scapulothoracic joint.^32^ This was done by calculating the Euler angles (e_1_: retraction/protraction angle, e_2_: lateral/medial rotation angle, e_3_: internal/external rotation angle) from $R_{scs}^{tcs}$ following a YXZ sequence.^32^

### Statistical analysis

The analyzed parameters were the 3D coordinates of IA, TS, and AA in the average scapula CS (9 parameters) and the Euler angles (e_1_: retraction/protraction, e_2_: lateral/medial rotation, e_3_: internal/external rotation) expressing the 3D scapular orientation around the axes of the scapulothoracic joint (3 parameters). Mean absolute deviation (ie, average distance between each measure and the mean) was expressed as mean, standard deviation, 95% confidence interval (CI) for each measure of each parameter. Intraobserver repeatability and interobserver reproducibility were assessed for each parameter using an intraclass correlation,^20^ respectively ${ICC}_{intra}$ and ${ICC}_{inter}$, as relative reliability metrics. Intraobserver repeatability represents in this study the degree of agreement between the results obtained by the same observer across the 3 manual positioning of each scapula. Interobserver reproducibility represents the degree of agreement among the 3 observers during manual positioning of each scapula. A single measure, 2-way, mixed model-type absolute ICC coefficients were used, that is, ICC (3,k).^27^ Variance components were estimated first with the *VCA* package (1.5.1).^7^ The total variance was defined as the class component variance sum: $\sigma_{total}^{2}=\sigma_{scapula}^{2}+\sigma_{observer}^{2}+\sigma_{measure}^{2}+\sigma_{residual}^{2}$ where $\sigma_{scapula}^{2}$, $\sigma_{observer}^{2}$, $\sigma_{measure}^{2}$, and $\sigma_{residual}^{2}$ are the scapula, observer, measure and residual variances, respectively. Following the methodology proposed by Chia and Sangeux,^4^ ICC estimates were obtained as follow:

(1) $$\begin{array}{c} {ICC\;}_{intra}=\frac{\sigma_{total}^{2}-\left(\sigma_{measure}^{2}+\sigma_{residual}^{2}\right)}{\sigma_{total}^{2}} \end{array}$$

(2) $$\begin{array}{c} {ICC\;}_{inter}=\frac{\sigma_{total}^{2}-\left(\sigma_{observer}^{2}+\sigma_{residual}^{2}\right)}{\sigma_{total}^{2}} \end{array}$$

ICC estimates were classified as poor (<0.50), moderate (0.50 to 0.75), good (0.75 to 0.90), and excellent (≥0.90).^15^ Standard error of measurement (SEM) was also quantified both for intraobserver repeatability and interobserver reproducibility as an absolute reliability metric and computed as follows:

(3) $$\begin{array}{c} {SEM\;}_{intra}=\sqrt{\sigma_{total}^{2}\times \left(1-{ICC\;}_{intra}\right)} \end{array}$$

(4) $$\begin{array}{c} {SEM\;}_{inter}=\sqrt{\sigma_{total}^{2}\times \left(1-{ICC\;}_{inter}\right)} \end{array}$$

The mean intraobserver repeatability and interobserver reproducibility 95% CI was computed as ±1.96 SEM.^20^ All computations related to the reliability analysis were performed in R 4.3.3 (R Project; University of Auckland, Auckland, New Zealand) and RStudio (version 2023.12.1 build 402; Posit, Boston, MA, USA).

## Results

### Population

One hundred patients were recruited in this study. Nineteen were excluded due to partial scapula view in CT scan images. Synthetized information of the resulting 81 patients is reported in Table II. Based on the worst ${ICC}_{inter}$ obtained for the position of the AA landmark along the Y axis of the scapula (${ICC}_{inter}=0.41$, Table II), the effect size was estimated at *d* = 0.32 using the following equation: $d=\left(1-{ICC}_{inter}\right)/\left(1+\left(k-1\right)\times {ICC}_{inter}\right)$.^27^ A post-hoc power analysis was then performed (G∗Power 3.1.9.7,^8^) and revealed a computed power of 0.81 (α = 0.05).

**Table II:** Mean absolute deviation, intraclass correlation, and standard error of measurement measured for each parameter.

Parameter	Unit	Mean absolute deviation			Intraobserver		Interobserver	
		Mean	Std	95% CI	ICC_intra_	SEM_intra_	ICC_inter_	SEM_inter_
AA_X_	mm	0.8	1.0	[0.7-0.9]	0.34	1.2	0.66∗	0.8
AA_Y_	mm	0.8	0.8	[0.7-0.8]	0.60∗	0.8	0.40	1.0
AA_Z_	mm	1.5	2.2	[1.3-1.6]	0.36	2.4	0.64∗	1.8
IA_X_	mm	0.7	0.6	[0.7-0.8]	0.59∗	0.7	0.41	0.8
IA_Y_	mm	0.5	0.5	[0.5-0.5]	1.00∗∗∗	0.5	1.00∗∗∗	0.6
IA_Z_	mm	1.1	1.0	[1.0-1.2]	0.99∗∗∗	1.0	0.98∗∗∗	1.5
TS_X_	mm	0.3	0.3	[0.3-0.3]	0.35	0.4	0.65∗	0.3
TS_Y_	mm	0.3	0.3	[0.3-0.3]	0.25	0.4	0.75∗∗	0.2
TS_Z_	mm	1.2	0.9	[1.2-1.3]	0.98∗∗∗	1.3	0.99∗∗∗	1.1
e_1_	deg	0.6	0.4	[0.5-0.5]	0.37	0.8	0.63∗	0.6
e_2_	deg	0.4	0.4	[0.4-0.5]	0.55∗	0.4	0.45	0.5
e_3_	deg	0.5	0.4	[0.5-0.5]	0.56∗	0.5	0.44	0.5

*AA*, acromial angle; *IA*, inferior angle; *CI*, confidence interval; *ICC*, intraclass correlation; *SEM*, standard error of measurement; *Std*, standard deviation; *TS*, trigonum spinae; *e*_*1*_, retraction/protraction angle; *e*_*2*_, lateral/medial rotation angle; *e*_*3*_, internal/external rotation angle.

Cotation: ∗: moderate ICC; ∗∗: good ICC; ∗∗∗: excellent ICC.

### Data distribution

**Figure 2:**
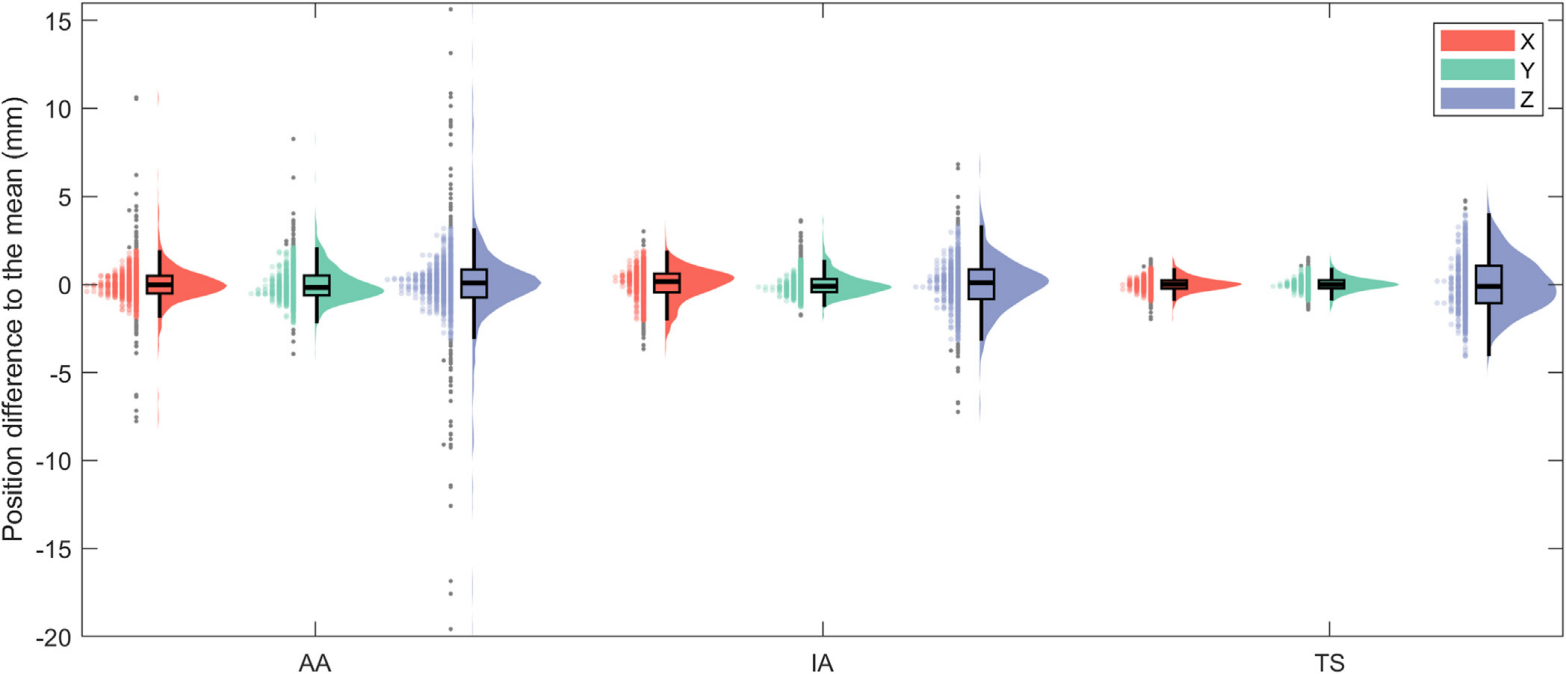
Distribution of the landmark coordinate difference to the mean for each anatomical landmark combining boxplots, kernel density, and half-violin plots.^14^ Outliers are reported as (*gray dots*). *AA*, acromial angle; *IA*, inferior angle; *TS*, trigonum spinae.

**Figure 3:**
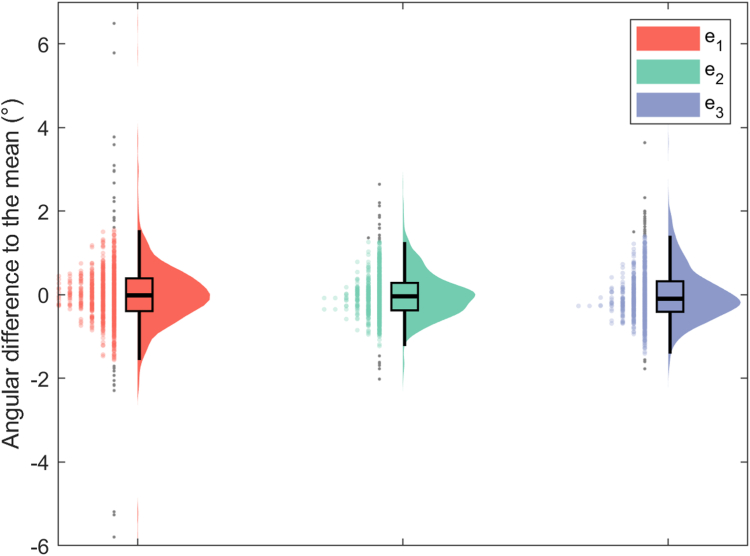
Distribution of scapular orientation difference to the mean around Euler angles combining boxplots, kernel density, and half-violin plots.^14^ Outliers are reported as (*gray dots*). *e*_*1*_, retraction/protraction; *e*_*2*_, lateral/medial rotation; *e*_*3*_, internal/external rotation.

Data distribution for each parameter is reported in Figures. 2 and 3 and in Table II. Concerning landmark position difference to the mean, the direction along Z axis demonstrated the largest deviations from the mean while the fourth quartile remained under 5 mm in all cases. Only AA demonstrated larger deviations compared to other landmarks along X and Y axes with numerous outliers for all three axes.

Concerning the scapular orientation difference to the mean, all the 3 Euler angles demonstrated similar deviations from the mean with the fourth quartile remains under 2° in all cases. It must, however, be noted that highest and most numerous outliers are observed for e_1_ angle.

### Intraobserver and interobserver repeatability

**Figure 4:**
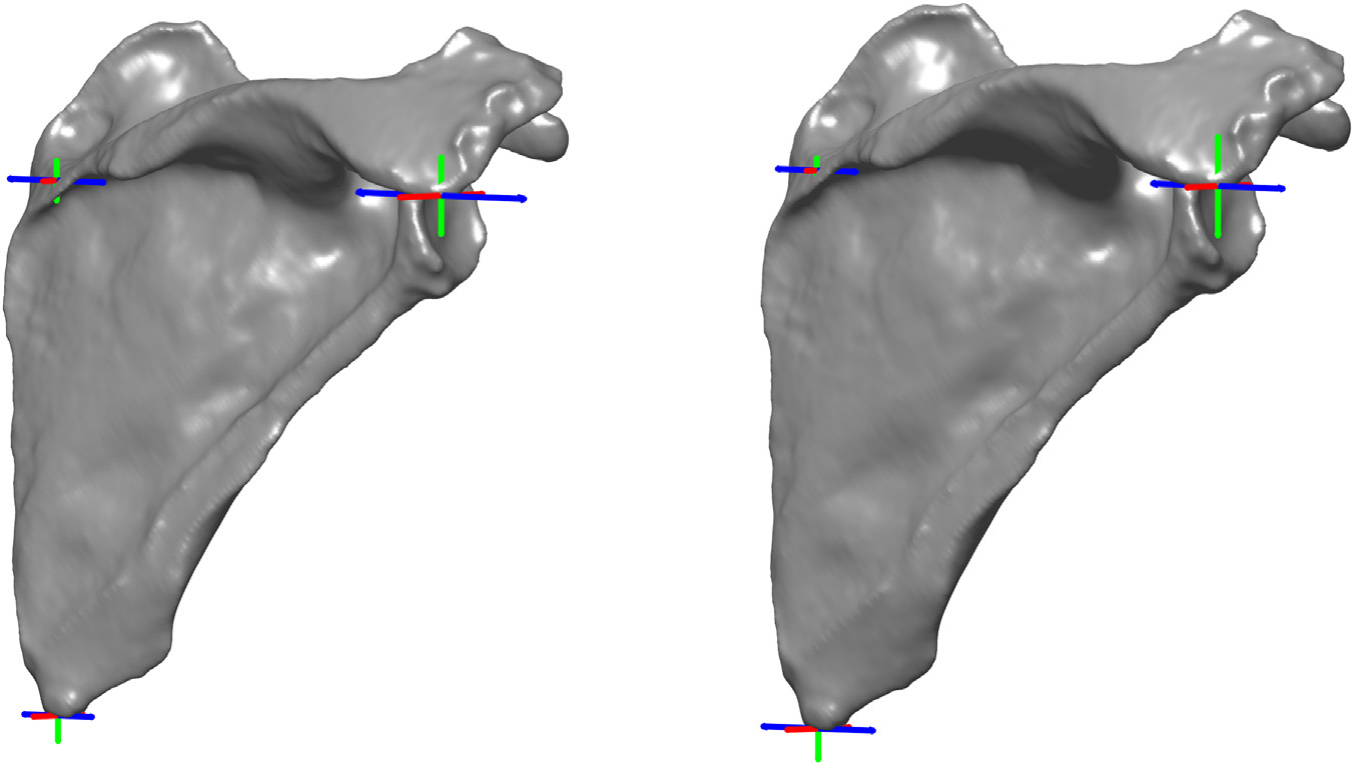
Illustration of the SEM observed for each anatomical landmark in each direction (X axis: *red*, Y axis: *green*, Z axis: *blue*) for intraobserver repeatability *(left)* and interobserver reproducibility *(right)*. For each direction, the SEM length (mm) is multiplied by 10 to improve the visualization and symmetrically reported from the mean position in each sense. *SEM*, standard error of measurement.

Intraobserver repeatability and interobserver reproducibility are reported as ICC and SEM values in Table II. Both ${SEM}_{intra}$ and ${SEM}_{inter}$ are also reported graphically in Figure 4 for each anatomical landmark in each direction. Intraobserver ICC ranged from poor to moderate for AA, moderate to excellent for IA, and poor to excellent for TS. However, low SEM ranging from 0.4 mm (TS along the X axis) to 2.4 mm (AA along the Z axis) were obtained for every coordinate of each anatomical landmark. This results in poor to moderate ICC for scapular orientation, with low SEM ranging from 0.4° (e_2_: lateral/medial rotation) to 0.8° (e_1_: retraction/protraction).

Interobserver ICC ranged from poor to moderate for AA, poor to excellent for IA, and moderate to excellent for TS. However, low SEM ranging from 0.2 mm (TS along the Y axis) to 1.8 mm (AA along the Z axis) was obtained for every coordinate of each anatomical landmark. This results in poor to moderate ICC for scapular orientation, with low SEM ranging from 0.4° (e_3_: internal/external rotation) to 0.6° (e_1_: retraction/protraction).

## Discussion

In this study, the intraobserver repeatability and interobserver reproducibility of 3D coordinates of 3 scapula landmarks obtained by manual positioning on conventional supine CT scan–based scapula surface models were assessed, as well as the intraobserver repeatability and interobserver reproducibility of the resulting 3D scapular orientation. The landmarks of interest were TS, IA, and AA, ie, the 3 landmarks proposed by the ISB recommendations^32^ to define the scapula CS and thus to define 3D scapular orientation. These landmarks were selected as they represent a relevant basis to apply bone registration approaches based on *in vivo* measurements using any sensor-based approach (and in most cases to any imaging-based approach). Noteworthy, articular landmarks, such as the glenoid center, which is being often used in imaging-based approaches,^12^ cannot be located by manual palpation and is thus not adapted to sensor-based approaches.

### Anatomical landmark positioning reliability

Relative reliability demonstrated poor to excellent ICC across observers and measures for analyzed anatomical landmark coordinates. These ICC values were particularly low concerning AA, which could mean that either this landmark is difficult to manually position consistently across different measures or by different observers. However, absolute reliability demonstrated SEM values remaining under 2 mm in most cases. This suggests that while there may be inconsistency in how the landmark positioning varies across observers and measures (as reflected by ICC values), the individual positionings themselves are very close to each other, leading to a low data dispersion, and so arguing for reliable measurements.

**Figure 5:**
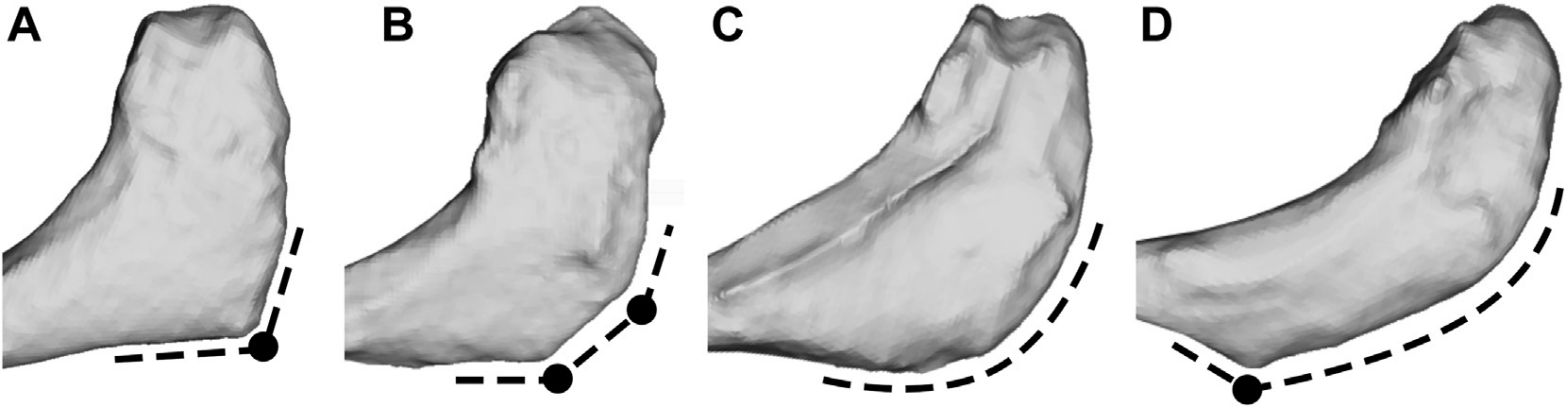
Illustration of most observed acromial shapes in the present dataset and impact on the acromial angle identification (*black dots*) represent obvious angles). From left to right: single clear angle from the lateral edge of the acromion (**A**), multiple angles from the lateral edge of the acromion (**B**), curved transition from the lateral edge of the acromion to the scapula spine without any clear angle (**C**), sometimes completed with an angle appearing at the first distal third of the scapula spine (**D**).

To the best of our knowledge, the reliability of AA manual positioning has never been assessed in the literature. It appears that the positioning of this anatomical landmark can be difficult due to the morphological variations observed in the present population (Fig. 5). While the ideal morphology can be defined as a single clear angle from the lateral edge of the acromion (Fig. 5, *A*), multiple angles can also be observed from the lateral edge of the acromion (Fig. 5, *B*), as well as a curved transition from the lateral edge of the acromion to the scapular spine without any clear angle (Fig. 5, *C*), sometimes completed with an angle appearing at the first distal third of the scapula spine (ie, near the insertion transition between lateral deltoid and posterior deltoid fibers) (Fig. 5, *D*).

These morphological variations mainly impact the medial–lateral position of AA (where the highest variability is observed) and secondarily the anterior–posterior position of AA. The impact on the present results was limited to a maximal SEM of 2.4 mm, probably linked to observer training and updated landmarks definition (Table I). Implementation of proposed definition or performance of a similar training work is recommended for further studies to avoid variability in landmark positioning process. The reliability of IA manual positioning was previously reported in the literature by Jacquot et al.^12^ In accordance with their study, the present results show greater variability on the medial–lateral position than in other directions. However, the mean and standard deviation of the mean absolute deviation have been reduced from 2.2 ± 1.9 mm (n = 86,^12^) to 1.1 ± 1.0 mm (n = 81, present results). Morphological variations can be observed at the distal part of the scapula with a possible flat shape suggesting multiple potential angles (see Fig. 4 in Jacquot et al^12^). The impact on the present results was limited to a maximal SEM of 1.5 mm, probably linked to observer training and to the updated landmarks definition as stated before (Table I). The reliability of TS manual positioning was previously reported in the literature by Jacquot et al^12^ and Gilliland et al.^10^ In the study of Jacquot et al,^12^ TS was located on the medial border of the scapula, at the junction area with the scapular spine. Due to the triangular shape of this area, a high variability was observed along the superior–inferior direction (6.3 ± 6.4 mm). In the study of Gilliland et al,^10^ TS was located at the midpoint of the triangular surface, following the ISB recommendations.^32^ With this definition based on observed estimation of a surface geometrical center, they reported a standard deviation of 8.1 mm. In the present study, the TS definition was changed after the training phase, from the original ISB definition to the apex of the root triangle, closest to the scapular spine (Table I) as proposed by Van Sint Jan.^28^ This results in a lower variability of TS, with a maximal SEM of 1.3 mm observed along the medial–lateral direction.

### Scapular orientation reliability

Relative reliability demonstrated poor to moderate ICC values across observers and measures for 3S scapular orientation. This suggests that there exists inconsistency between the measurements, which indicates a potential lack of reliability in defining scapular orientation. In particular, further outliers were observed around e_1_ (retraction/protraction) with an angular difference to the mean up to 4°. However, absolute reliability demonstrated SEM values remaining under 1° in all cases. This suggests that while there may be inconsistency in how the scapular orientation varies across observers and measures (as reflected by ICC values), the individual measurements themselves are very close to each other, leading to a low data dispersion, and so arguing for reliable measurements.

The reliability of scapular orientation based on landmarks defined on imaging data was previously reported by Zwingenberger et al^33^ on 2-dimensional radiographs. The present results show a lower data dispersion on the lateral–medial rotation of the scapula (0.4 ± 0.4° vs. 2.0 ± 1.0° using the medial edge of the scapula^33^). The limited impact of the anatomical landmark positioning on the 3D scapular orientation can be explained by the scapula CS construction. Indeed, landmark coordinate data dispersion was primarily observed along the medial–lateral axis. However, moving AA and/or TS along this direction has no impact on the definition of the medial–lateral axis. Similarly, moving AA, IA, and/or TS along this direction has no impact on the definition of the anterior-posterior axis as the plan they define does not change. The propagation error of anatomical landmark positioning on the 3D scapular orientation on the surface model (95% CI up to 1.0°) must be considered together with the error observed on the scapular orientation during *in vivo* measurements (95% CI up to 3.4° using biplanar X-rays^3^ and mean error up to 2.0° using a calibrated stylus or anatomical palpator^25^). Overall, the resulting 3D scapular orientation data dispersion is close to the intraoperative baseplate positioning data dispersion in rTSA, ie, 5.9 ± 3.5° and more using a conventional approach, and 1.9 ± 1.9° and more using navigation.^13^^,^^31^

### Limitations

Proposed approach is subject to following limitations. First, a bone surface model is required to defined anatomical landmarks and later to perform the bone registration method. This bone surface model is obtained by segmentation of CT scan images, ie, an ionizing approach. Still, such imaging is becoming standards in rTSA planification procedures. Thus, this does not imply any additional patient irradiation. Second, the reliability of bone surface models is unknown. As being performed by the implant manufacturer, these data cannot be assessed at this stage. However, the related variability may impact the anatomical landmark positioning and thus the scapular orientation. Third, the impact of anatomical landmark positioning on glenoid orientation (ie, version and inclination) was not assessed. Such information represents a crucial factor in surgical planning. However, positioning of additional landmarks would have been necessary to define the glenoid location in the scapula surface model, which may introduce another source of error. Furthermore, considering the scapula bone as a rigid structure, the orientation reliability reported in the present study includes the glenoid (that can be preserved or abnormal), even if further research would be needed to report the impact of landmark positioning on glenoid orientation around its major (version angle) and minor (inclination angle) axes. Fourth, the limited relative reliability (ICC) reported in the results indicates that care must be taken to ensure consistency in how these measurements are performed. This could be improved for example through a more rigorous training of operators and by still improving the landmark definitions.

## Conclusion

This study demonstrates that manual positioning of the 3 scapula anatomical landmarks recommended by the ISB can be performed in a reliable manner across measures and observers on surface bone models obtained from CT scan images. However, a clear and standardized definition of these landmarks is needed to ensure consistency across measurements.

## Acknowledgment

The authors would like to thank the patients for their contribution to this study.

## Disclaimers:

Funding: The authors did not receive any financial remuneration for thy study.

Conflicts of interest: The authors, their immediate families, and any research foundations with which they are affiliated have not received any financial payments or other benefits from any commercial entity related to the subject of this article.
